# Staged Closure of Giant Omphalocele using Synthetic Mesh

**Published:** 2014-09-01

**Authors:** Lalit Parida, Kamalesh Pal, Hussah Al Buainain, Hossam Elshafei

**Affiliations:** 1 Department of Pediatric Surgery, GMC Hospital, Ajman, United Arab Emirates; 2 Division of Pediatric Surgery, Department of Surgery, University of Dammam, King Fahd Hospital of the University, Al Khobar, Saudi Arabia

**Keywords:** Giant omphalocele, Staged closure, Silo, Synthetic mesh

## Abstract

Giant omphalocele is difficult to manage and is associated with a poor outcome. A male newborn presented to our hospital with a giant omphalocele. We performed a staged closure of giant omphalocele using synthetic mesh to construct a silo and then mesh abdominoplasty in the neonatal period that led to a successful outcome within a reasonable period of hospital stay.

## INTRODUCTION

A giant omphalocele is very difficult to treat at birth because of viscero-abdominal disproportion. Primary closure is usually not possible in these cases and many innovative techniques have been described in literature.[1-4] We report a successful utilization of a synthetic mesh in the staged closure of giant omphalocele in the neonatal period.

## CASE REPORT

A full term male neonate with a birth weight of 3.075 kg was born by spontaneous vaginal delivery to a primigravida. The baby was noticed to have multiple congenital anomalies; a giant omphalocele, cleft lip/palate, left anophthalmia, and congenital heart disease. On examination, the baby had a giant omphalocele with an approximate 10 x 10 cm anterior abdominal wall defect through which liver and most of the bowel loops had herniated into an intact sac. During surgery the amnion sac was excised and the bowel loops were reduced into the abdomen. At the end of maximal reduction of contents, it was observed that the major part of the liver could not be accommodated into the abdomen. Hence, a silo mesh was placed around the liver. We constructed the silo using a PhysiomeshTM mesh (Ethicon, Johnson and Johnson International, USA) sandwiched between two layers of OpsiteTM (Smith and Nephew Inc, St. Petersburg FL, USA). The baby was transferred back to the neonatal intensive care unit (NICU) and electively put on mechanical ventilation.

A daily plan of sequential reduction of silo content was initiated from second post-operative day. We were able to achieve a daily one centimeter height reduction of the silo in the NICU under sterile conditions (Fig.1). Maximal reduction of silo content was attained by seventh post-operative day. The baby was taken to the operation theatre on eighth day and the liver was reduced into the abdominal cavity. The margins of the muscular aponeurotic plane could not be approximated to the midline at an acceptable intra-abdominal pressure and hence the residual defect was closed with a 5 x 5 cm PhysiomeshTM patch (without any OpsiteTM )and midline skin closure was achieved. 

**Figure:**
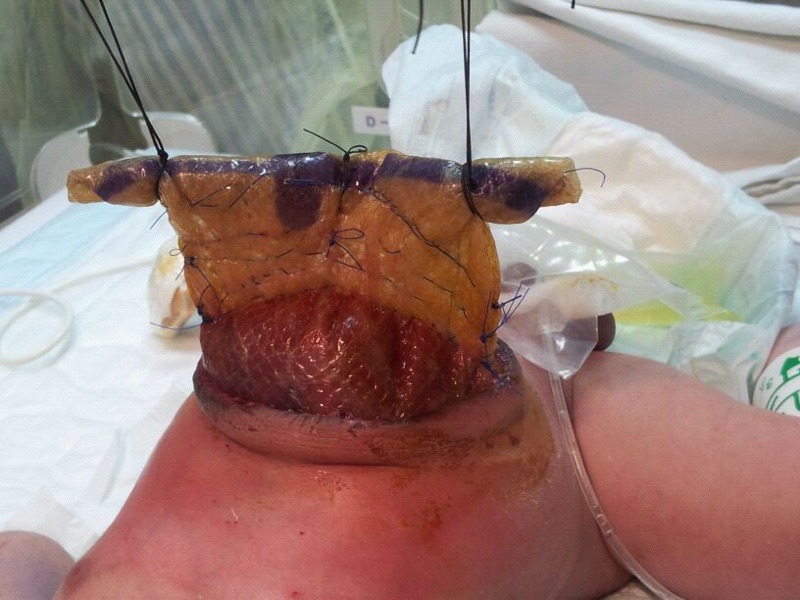
Figure 1:Daily sequential reduction of synthetic mesh silo content

A seroma developed above the mesh which subsided with aspirations only. Cutaneous hyperemia was noticed overlying the mesh on second post-operative day which subsided spontaneously by seventh post-operative day (Fig.2). The aspirated fluid from the seroma and wound swab revealed no growth of organisms. The baby was weaned off from the sedation and mechanical ventilation on the third post-operative day. Oral feeding was established and he was discharged with a healed abdominal wound on the tenth post-operative day. The total duration of hospitalization was 18 days. At three months follow up, the baby was found to have a healthy anterior abdominal wall with no ventral hernia, granuloma or sinus.

**Figure:**
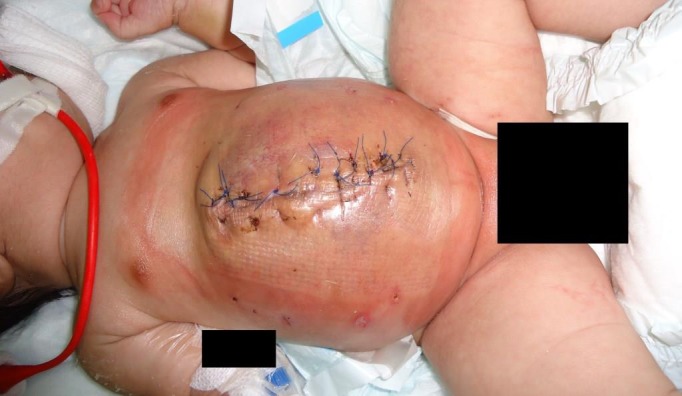
Figure 2:Complete closure of giant omphalocele

## DISCUSSION

The treatment of giant omphalocele at birth is a complex clinical problem and usually involves either primary closure, staged closure, or an initial conservative or non-operative treatment.[2] We adopted a staged operative approach by constructing the silo with PhysiomeshTM and OpsiteTM; while during the second surgery, closure of the abdominal fascial defect needed only the PhysiomeshTM.

The silo used was a flexible synthetic mesh consisting of a polypropylene mesh laminated between two polyglecaprone-25 films. This transparent silo mesh allowed to view the liver while reducing it. The polyglecaprone-25 layer improves the handling by facilitating suture placement and has enough memory for easy deployment around the abdominal fascial defect. This layer also provides a physical barrier between the polypropylene mesh and adjacent tissue, thus preventing adhesions. The OpsiteTM provides a transparent waterproof layer which is permeable to water vapor and oxygen but impermeable to micro-organisms.

Preformed spring loaded silo bags have been used in the staged management of abdominal wall defects, especially in gastroschisis and ruptured omphalocele. Its limitations include local unavailability and presence of a stainless steel spring at its open end which can cut through its silicone coating and injure the liver or bowel. It cannot be utilized in omphaloceles with an intact sac as the spring loaded ring needs to be under the fascial defect.[5] Silastic sheets have been used in closure of abdominal wall defects but it is not stretchable and does not integrate into local tissue. Moreover, sutures can cut through the silastic material resulting in silo-fascial dehiscence.[6] Meshes made up of Teflon (Gore-Tex) and bovine pericardium (Tutomesh) have been described for primary closure of omphaloceles, but not for staged closure like in our patient. Gore-Tex patches have been associated with persistent infection requiring removal.[4]

Once staged closure has been undertaken, a controlled and committed policy for daily reduction is done to achieve the complete reduction of contents within a five to seven days period in order to prevent silo-fascial dehiscence and sepsis. However, in a subset of giant omphalocele, staged reduction may still be incomplete at the end of this timeline or silo-fascial dehiscence may ensue requiring the surgeon to undertake excision of silo and attempt abdominal closure. Use of composite mesh like PhysiomeshTM allows for abdominoplasty in the neonatal period which is technically simple and has few but manageable complications (seroma, sterile inflammatory response). The seroma observed in our patient is an expected transient non-infective inflammatory reaction that does not interfere with the mesh integration into contiguous tissue. Besides this and the transient local cutaneous hyperemia, there was no other complication. The neonate was able to feed orally within a few days after surgery. In conclusion, we performed complete repair of giant omphalocele in the neonatal period within an acceptable period of hospital stay using this technique.

## Footnotes

**Source of Support:** Nil

**Conflict of Interest:** None declared

